# AI-driven immunotherapy: synergizing with radiotherapy to reconfigure the tumor microenvironment and treatment landscape

**DOI:** 10.3389/fphar.2025.1747638

**Published:** 2026-01-06

**Authors:** Dongling Gu, Yi Feng, Hongyan Li

**Affiliations:** 1 Shantou University, Shantou, China; 2 Department of Anesthesiology, The Affiliated Traditional Chinese Medicine Hospital, Southwest Medical University, Luzhou, Sichuan, China; 3 Luzhou Key Laboratory of Research for Integrative on Pain and Perioperative Organ Protection, Luzhou, Sichuan, China

**Keywords:** artificial intelligence, biomarkers, immunotherapy, radiomics, radiotherapy, tumor immune microenvironment

## Abstract

Immunotherapy plays a crucial role in cancer treatment, but its efficacy varies among patients, with some showing suboptimal responses. Recent studies indicate that radiotherapy not only kills tumor cells locally but also induces immunogenic cell death and modulates the tumor immune microenvironment, acting like an “*in situ* vaccine.” This provides a strong biological basis for combining radiotherapy and immunotherapy. However, challenges remain, including individual variability in responses, complex treatment regimens, and overlapping toxicities. Artificial intelligence (AI), especially through machine learning, presents new solutions by processing high-dimensional multi-omics data. This article explores how AI enhances radiotherapy and immunotherapy combinations by optimizing synergistic effects, developing predictive biomarkers, and elucidating the regulatory mechanisms of radiotherapy on the immune microenvironment, while also discussing future directions for AI in oncology.

## 1 Introduction

Immunotherapy has taken on an important role in cancer treatment, however, its efficacy varies significantly among different patients, with some exhibiting suboptimal responses. Since the last century, radiotherapy has been a crucial weapon against cancer. Its core principle involves using high-energy radiation to directly damage tumor cell DNA, thereby inhibiting proliferation and inducing apoptosis (Bo et al., 2016; Dörthe and William, 2015). Beyond its established role in direct tumor control, radiotherapy demonstrates significant immunomodulatory effects that form the foundation for synergistic combination with immunotherapy. Within this classic framework, the goal of treatment is to maximize local tumor control while minimizing damage to surrounding normal tissues. However, a remarkable clinical observation—the “abscopal effect,” where shrinkage of non-irradiated distant metastases occurs after local irradiation of a lesion, suggests that radiotherapy’s impact extends far beyond local effects (María et al., 2018; Yuwen et al., 2024). The immunological mechanisms underlying this phenomenon have been gradually uncovered: radiotherapy induces immunogenic cell death, releasing signals such as tumor-associated antigens, damage-associated molecular patterns, and high mobility group box 1 (HMGB1). This process effectively creates an “*in situ* vaccine” within the body. These signals promote the maturation and antigen presentation by dendritic cells, which in turn activate tumor-specific T cells, potentially triggering a systemic anti-tumor immune response (Katalin and Géza, 2013). Concurrently, cancer immunotherapy, particularly immune checkpoint inhibitors (ICIs), has achieved revolutionary breakthroughs, offering hope for long-term survival in advanced patients. However, the efficacy of ICI monotherapy remains high only in certain cancer types, and a significant number of patients experience primary or acquired resistance. Radiotherapy’s ability to convert immunologically “cold” tumors into “hot” ones by enhancing antigen presentation and T-cell recruitment makes its combination with immunotherapy a highly promising strategy. Consequently, the combination of radiotherapy and immunotherapy is regarded as a highly promising strategy (Jiang et al., 2024; Xu et al., 2025).

**FIGURE 1:**
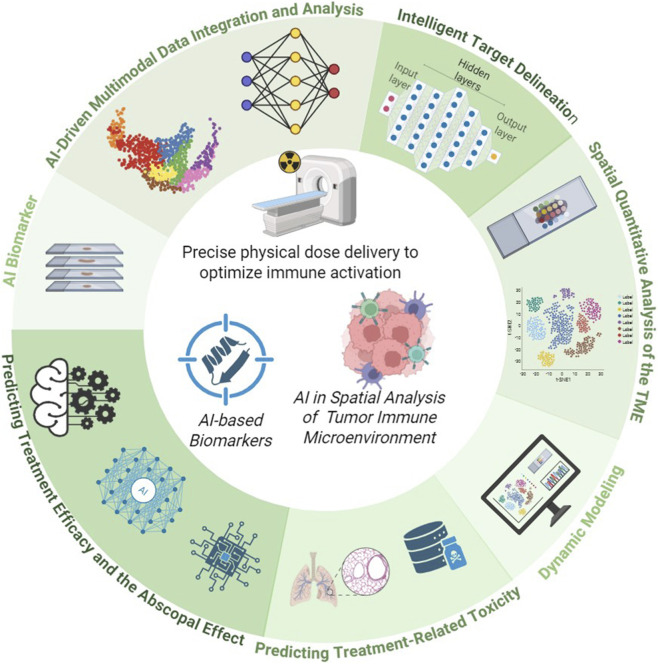
The framework for AI-driven synergistic empowerment of radiotherapy and immunotherapy.

However, this promise is underpinned by substantial complexity. First, radiotherapy exerts a “double-edged sword” effect on the immune system: while it can activate immune responses, it may also enhance immunosuppression by recruiting regulatory T cells and inducing myeloid-derived suppressor cells. Second, the optimal combination strategy, encompassing radiotherapy timing, dose, fractionation, and target volume, remains elusive. Furthermore, combination therapy can lead to overlapping toxicities, such as the concurrent onset of severe radiation pneumonitis and immune-related pneumonitis. To address these high-dimensional, nonlinear complexities, artificial intelligence (AI) offers innovative solutions. Over the past decade, rapid advances in computational power and data storage have driven the development and application of cutting-edge AI technologies in radiological image processing. Although computerized imaging techniques have been utilized in radiology since the 1960s (Li et al., 2025; Maliazurina et al., 2025), the field is now experiencing a period of explosive growth, particularly within oncologic imaging. AI, especially deep learning, can identify patterns imperceptible to the human eye from vast amounts of medical images, genomic, and pathological data. This capability enables the construction of predictive models and provides data-driven insights for individualized treatment decisions (Hideyuki and Keiichi, 2020). Specifically, AI demonstrates unique potential in optimizing combination therapy through pharmacological modeling and patient stratification. By integrating multi-omics data with clinical pharmacokinetic/pharmacodynamic parameters, AI models can simulate and predict the *in vivo* dynamic processes of ICIs combined with radiotherapy under various timing and dosing regimens. For example, machine learning-based models can quantify radiotherapy-induced changes affecting ICI distribution and immune activation, thereby identifying optimal combination strategies that maximize synergy while minimizing toxicity. Furthermore, by analyzing dynamic characteristics of the tumor immune microenvironment, AI can identify patient subgroups most responsive to specific ICI classes, enabling personalized treatment design. This review systematically elaborates on how AI empowers radiotherapy across three dimensions: achieving precise physical dose delivery to optimize immune activation, serving as predictive biomarkers for treatment efficacy, and dynamically decoding the regulatory mechanisms of radiotherapy on the immune microenvironment (Figure 1). We will comprehensively examine how AI acts as a core enabling technology, bridging the physical world of radiotherapy and the biological world of immunity, thereby propelling cancer therapy into a new intelligent era.

## 2 AI as a bridge to synergy between radiotherapy and immunotherapy

Precision is the cornerstone of radiotherapy and a prerequisite for its effective interplay with the immune system. AI has played a revolutionary role in enhancing the physical accuracy of radiotherapy, thereby laying a solid foundation for subsequent immune modulation.

### 2.1 AI-driven target contouring

Traditional target delineation relies on manual contouring by radiation oncologists, which is time-consuming and susceptible to subjective influences, leading to significant inter-observer variability. AI technologies, particularly convolutional neural networks (CNNs), enable automatic, precise, and efficient delineation of both gross tumor volume (GTV) and clinical target volume (CTV). For example, a CNN-based method for automatic segmentation of intra-prostatic tumors on PSMA PET images demonstrated high sensitivity and specificity, providing a robust technique for primary prostate cancer treatment (Dejan et al., 2020). Another study developed a CNN architecture for pancreatic GTV segmentation using multiparametric MRI, achieving performance comparable to experts and offering a framework for MR-guided online adaptive radiotherapy (Ying et al., 2020). This automation improves workflow efficiency, reduces errors, and promotes standardization. Intratumoral heterogeneity leads to variations in radiotherapeutic response across regions. AI enables delineation of “biological target volumes” or “immunological target volumes” by extracting quantitative features from conventional imaging (e.g., CT, PET, or MRI) to identify subregions with distinct biological characteristics, such as hypoxic or proliferative zones (Akifumi et al., 2023). Multiparametric magnetic resonance imaging (mp-MRI) and innovative techniques like synthetic MRI address limitations of individual imaging methods and support personalized strategies. For instance, a lactate-related prognostic signature (LPS) model predicted survival and treatment responses in esophageal squamous cell carcinoma, with low-LPS patients showing better outcomes and high-LPS patients exhibiting more genetic alterations (Chuting et al., 2025). Knowledge-based planning (KBP) automates traditional treatment planning by generating dose distributions, dose-volume histograms (DVHs), and constraints for planning target volumes (PTVs) and organs at risk (OARs) (Yi et al., 2024). Deep learning has advanced KBP, with studies using U-Net and ResUNet architectures to predict dose distributions for prostate and head and neck cancers (Elizabeth et al., 2020; Dan et al., 2019; Jiawei et al., 2018). Additionally, a linear mixed-effects model compared brain age changes before and after whole-brain radiotherapy (WBRT), revealing accelerated aging in irradiated patients and highlighting the protective role of hippocampal avoidance (Nik et al., 2023). In summary, AI enhances radiotherapy precision and efficiency while enabling personalized therapy and deeper insights into the immune microenvironment, signaling a transformative shift in oncology.

### 2.2 AI-driven integration and analysis of multimodal data

AI enables the integration of multimodal data from diverse sources—including imaging, genomics, and clinical information—to provide comprehensive patient profiles. For instance, a 3D CNN-based method for automatic delineation of head and neck cancer targets demonstrated that multimodal auto-contouring is more effective and consistent than unimodal approaches (Heleen et al., 2023). Another team developed a multimodal framework combining radiological and histopathological images to characterize lung adenocarcinoma, where radiopathological features showed strong prognostic power for postoperative decisions (Huan et al., 2025). Furthermore, AI shows potential to transform upper gastrointestinal disease management. Deep learning improves diagnostic accuracy, reduces interobserver variability, and enhances early cancer detection, lesion characterization, and invasion depth prediction. Future applications may include real-time endoscopic guidance and optimized biopsy decisions, improving both outcomes and cost-effectiveness (Alanna et al., 2025). Such data fusion supports personalized treatment strategies and deeper understanding of tumor biology. AI analysis of multi-omics data can identify potential biomarkers, with multimodal methodologies expected to yield novel biomarkers advancing precision oncology (Kevin et al., 2021). The Swirl-Multimodal and Multi-region Data Fusion Framework (SMuRF) exemplifies this approach, integrating CT features from primary tumors and lymph nodes with whole-slide pathology images to predict survival and tumor grade in HPV-associated oropharyngeal cancer. SMuRF demonstrated superior performance (C-index = 0.81) and emerged as an independent prognostic biomarker, outperforming unimodal models (Bolin et al., 2025). These AI-driven approaches not only predict treatment response but also inform selection of radiotherapy and immunotherapy strategies, enhancing therapeutic precision. By providing real-time, data-driven decision support, AI systems help clinicians design more effective treatment regimens—ultimately improving patient survival and quality of life.

### 2.3 AI in optimizing immunoradiotherapy

In recent medical research, artificial intelligence (AI) has demonstrated extensive potential for optimizing immunotherapy and radiotherapy. Through data analysis and machine learning algorithms, AI can assist doctors in formulating more precise, personalized treatment plans, thereby improving patient outcomes. In the field of immunotherapy, AI can analyze patients’ genomic data, tumor characteristics, and prior treatment responses to predict the efficacy of specific immunotherapeutic drugs. For example, researchers have used AI algorithms to analyze genomic data from a large number of cancer patients, identifying biomarkers associated with responses to immune checkpoint inhibitors, such as PD-1/PD-L1 inhibitors (Sahu et al., 2025). These findings indicate that AI can significantly enhance the identification of patients likely to benefit from immunotherapy, paving the way for more personalized treatment recommendations. Additionally, the application of AI in radiotherapy is gaining increasing attention. Traditional radiotherapy planning often relies on physicians’ experience and manual adjustments, which may lead to inefficiencies or increased side effects. In this regard, AI technology can analyze patients’ imaging data to automatically generate radiotherapy plans and optimize radiation dose distribution. This approach aims to maximize protection of healthy tissues while delivering higher radiation doses to tumors (Chelliah et al., 2024). For instance, a study has shown that radiotherapy plans generated using deep learning algorithms achieve higher precision and reduce radiation exposure to normal tissues compared to conventional methods (Kashyap et al., 2025).

## 3 AI-based biomarkers

Predicting which patients will benefit from radiotherapy, particularly from combined radiotherapy and immunotherapy, is crucial for achieving precision medicine. Artificial intelligence is emerging as a powerful tool for discovering and defining novel biomarkers.

### 3.1 AI-discovered biomarkers

AI has played a pivotal role in extracting valuable information from complex, multi-dimensional datasets (Su et al., 2025). In translational oncology, AI-driven analytical approaches have uncovered cellular state signatures (Avishai et al., 2023), interaction mechanisms within tumor tissues (Chia-Kuei et al., 2024), and their influence on treatment responses (Beth et al., 2020). A biomarker is defined as “a defined characteristic that is measured as an indicator of normal biological processes, pathogenic processes, or responses to an exposure or intervention, including therapeutic interventions” (Sehhoon et al., 2022). In oncology, biomarkers are applied across a broad spectrum—from preventive uses such as assessing cancer susceptibility or risk, to guiding high-level clinical decision-making, among which prognostic and predictive biomarkers hold the greatest clinical significance. Currently, commonly used prognostic biomarkers in oncology are predominantly molecular assays, such as Oncotype DX and MammaPrint in breast cancer (Daniel and Ira, 2017), and the Decipher test in prostate cancer (Sanjeev et al., 2018). These tests are based on complex multi-gene expression signatures. Although these genomic assays have been incorporated into the National Comprehensive Cancer Network (NCCN) guidelines and are used in routine clinical practice, their widespread application and utility for continuous monitoring throughout treatment remain limited due to high costs and the requirement for invasive tumor tissue sampling. In contrast, AI-based predictive or prognostic imaging biomarkers offer distinct advantages. These biomarkers can be derived from routine clinical imaging scans, making them non-invasive, non-destructive, rapid, easily standardized, and relatively low in cost (Joel et al., 2018). They integrate seamlessly into existing clinical workflows, similar to AI-based pathological biomarkers (Matthew et al., 2019), while retaining the benefit of being non-invasive (Aurélien et al., 2020; Jiang et al., 2023). Moreover, they enable characterization of the entire three-dimensional tumor volume, thereby mitigating sampling bias inherent in heterogeneous tumor biopsies (Edward et al., 2015), and are capable of detecting dynamic changes in the tumor microenvironment (TME). For example, researchers have developed an AI-based whole-slide image analysis system for evaluating tumor-infiltrating lymphocytes (TILs) in the TME. This system classifies three immune phenotypes: inflamed, immune-excluded, and immune-desert. AI-driven spatial analysis of TILs was correlated with tumor response and progression-free survival in patients with advanced non-small cell lung cancer (NSCLC) treated with ICIs, suggesting its potential as a complementary biomarker to pathologist-determined tumor proportion score (TPS). Thus, AI-based predictive biomarkers hold immense and expanding potential for future applications.

### 3.2 Predicting therapeutic efficacy and abscopal response

Over recent decades, big data has become indispensable in clinical oncology, encompassing medical images, genetic sequences, laboratory results, and pathological slides (Qiang et al., 2025). These data support diagnosis, treatment evaluation, outcome prediction, and mechanistic insights. Cancer research increasingly integrates large-scale datasets, with AI and deep learning now widely employed in precision medicine to analyze multi-scale data and build decision-support systems (Ziqi et al., 2019). In precision immunotherapy, AI deciphers immune and cancer features to optimize diagnosis, efficacy evaluation, and prognosis prediction. AI models automatically extract insights from multi-omics data, aiding immunotherapy decisions. Across cancer types, AI has achieved diagnostic performance comparable to or surpassing human experts (Scott Mayer et al., 2020; Babak et al., 2017), enabling applications in prognostic assessment, treatment response prediction, and discovery of imaging phenotypes linked to demographic and genotypic factors (Li et al., 2025). Although theoretically appealing, the abscopal effect occurs in only about 10% of cases, making early identification of potential responders a critical challenge. Conventional methods often fail to predict its occurrence, necessitating new tools. Radiomics combined with deep learning offers a promising solution by extracting quantitative features from pre-treatment CT or PET images and training machine learning models (e.g., support vector machines, random forests, neural networks). For example, a deep learning model analyzing baseline lung cancer CT images predicted response to immune checkpoint inhibitors—a strategy applicable to predicting abscopal effects post-radiotherapy. Radiomics, emerging around 2012, mines high-dimensional imaging features to support diagnosis and treatment planning (Philippe et al., 2012; Hugo, 2016; Chi et al., 2024). Classical machine learning techniques like SVM have been used for nasopharyngeal carcinoma staging, treatment response prediction, and radiation injury biomarker identification (Lina et al., 2019; Bin et al., 2017; Lu et al., 2019). Deep learning-based radiomic models also predict durable clinical benefit from ICI-based conversion therapy in hepatocellular carcinoma, revealing immune-related mechanisms (Zijian et al., 2025). Integration with genomics—radiogenomics—enables more comprehensive tumor biological assessment (Song et al., 2024). AI now facilitates multi-omics integration, including imaging, genomic (e.g., tumor mutation burden), transcriptomic (e.g., interferon-gamma signaling), proteomic (e.g., PD-L1), and clinical data (e.g., performance status). Holistic analysis builds powerful predictive models, creating a “digital twin” for evaluating immune baseline and response potential beyond single biomarkers. Radiomics complements molecular biomarkers with non-invasive spatial information and has been validated alongside whole-genome epigenetic modifications as potential prognostic biomarkers (Zhi et al., 2024). One study developed a prognostic model using six CpG probes from endoscopic biopsies via whole-genome methylation analysis (Wei-Lun et al., 2016). As the “omics era” advances, falling costs and improved reproducibility will further promote molecular data collection and integration across biological levels.

### 3.3 Predicting the risk of treatment-related toxicity

The combination of radiotherapy and immunotherapy offers potential synergistic effects but also increases the risk of overlapping toxicities. For instance, while thoracic radiotherapy often causes radiation pneumonitis (RP), immune checkpoint inhibitors can induce checkpoint inhibitor pneumonitis (CIP). When combined, these treatments significantly elevate the risk of severe pneumonitis, underscoring the need to identify high-risk patients (Qiang et al., 2025). Artificial intelligence models are advancing toxicity prediction. Lewinson et al. developed an artificial neural network integrating tumor characteristics, treatment parameters, medical history, neutrophil-to-lymphocyte ratio (NLR), lactate dehydrogenase (LDH), ECOG performance status, and tumor stage to predict ICI-related skin adverse events (Lewinson et al., 2021). In a study of 126 NSCLC patients receiving combined therapy, Qiu et al. identified an 11-feature CT signature that effectively differentiated CIP from RP, achieving AUCs of 0.891 (empirical) and 0.896 (model-calculated) (Qingtao et al., 2022). Bioinformatics analysis of inflammatory pathways and standardized adverse event databases, such as that established by Wang et al., further support mechanism elucidation and ICI safety optimization (QuanQiu and Rong, 2019). Although toxicity prediction remains nascent, progress is accelerating. AI models incorporating dose-volume histograms, baseline pulmonary imaging, and clinical factors enable accurate prediction of severe radiation pneumonitis risk. One machine learning study combined inflammatory cytokines with clinical variables to predict grade ≥2 RP before radiotherapy, providing a clinical decision-making tool (Yu et al., 2019). By prospectively identifying high-risk patients, clinicians can adjust radiation doses or modify treatment schedules to minimize adverse effects. Thus, AI-driven toxicity prediction not only enhances patient safety but also optimizes combination therapy strategies, ultimately improving treatment experience and quality of life.

## 4 AI in spatial analysis of the radiotherapy-related tumor microenvironment

Understanding how radiotherapy dynamically remodels the tumor microenvironment provides the biological foundation for optimizing combination therapy strategies. Artificial intelligence, particularly when applied to the analysis of digital pathology images, offers a powerful window into this complex process.

### 4.1 Digital pathology for TME spatial quantification

Traditional pathological analysis relies on visual inspection and semi-quantitative assessments, limiting accurate characterization of cellular spatial relationships. Digital pathology advances have enabled whole-slide imaging (WSI) of H&E and multiplex immunofluorescence stains, while convolutional neural networks now facilitate efficient nucleus segmentation, identification, and classification. This significantly improves both analytical efficiency and quantitative accuracy (Weicai et al., 2025). The transition to WSI offers superior resolution, streamlined storage, and enhanced data transmission compared to conventional microscopy. The tumor microenvironment critically influences tumor behavior and treatment response through immune cell infiltration, cytokine signaling, and stromal-vascular interactions (Karin and Johanna, 2023; Chao et al., 2021). AI now integrates radiological, histopathological, and genomic data to comprehensively characterize the TME, creating new strategies for evaluating tumor biology and predicting outcomes (Jana et al., 2022; Lili et al., 2021). High-dimensional features from digital pathology and medical imaging correlate strongly with immune activity, angiogenesis, and stromal interactions, proving vital for predicting chemotherapy and immunotherapy responses (Dexin et al., 2022; Shuo et al., 2022; Shaoxu et al., 2023). Both radiomics and pathomics demonstrate significant potential in immunotherapy prediction (Zhen et al., 2024). In clinical implementation, AI models using H&E-stained digital slides provide tissue-preserving diagnostic solutions. One study developed a computational EGFR biomarker model from 8,461 lung adenocarcinoma slides, achieving clinical-grade accuracy (AUC = 0.890) and reducing required molecular tests by 43% (Gabriele et al., 2025). Another team created RlapsRisk BC for early breast cancer, predicting 5-year metastasis-free survival (C-index 0.81) and improving risk stratification beyond conventional clinicopathological factors (Garberis et al., 2025). AI enables precise quantification of cellular densities and spatial architecture in tumor sections, measuring distances between cytotoxic T cells and tumor cells to distinguish immune phenotypes (“immune-excluded,” “immune-desert,” “immune-inflamed”). This spatial analysis reveals radiotherapy-induced TME modifications and provides insights into immune status dynamics. In NSCLC, AI-driven spatial TME analysis assessed dynamic changes and acquired resistance to EGFR-TKIs, predicting ICI therapy response (Yeong Hak et al., 2025). For iCCA, a spatial multi-omics approach using just 1 mm^2^ tissue accurately predicted outcomes and immunotherapy response (Libing et al., 2025). Where spatial transcriptomics remains clinically limited, deep learning systems infer TME characteristics from histology images to support prognosis prediction (Ruitian et al., 2024). By analyzing pre- and post-radiotherapy changes in immune cell distribution and spatial organization, AI captures high-dimensional quantitative spatial data that elucidates radiotherapy’s immunomodulatory mechanisms. Through precise TME analysis and dynamic prediction, AI fundamentally supports personalized treatment strategy development in combined radiotherapy and immunotherapy.

### 4.2 Dynamic modeling and mechanistic exploration

Conventional analyses often focus on static descriptions and struggle to capture dynamic immune microenvironment changes in real time. In contrast, artificial intelligence constructs dynamic models by integrating longitudinal imaging, pathology, and blood-based biomarkers—such as circulating tumor DNA and cytokines—revealing the evolving trajectory of the tumor microenvironment after radiotherapy. Through machine learning analysis of spatial tumor-infiltrating lymphocyte distributions, AI elucidates cell-type interactions and host-tumor interplay, predicting immune response nature and therapeutic efficacy (Tze et al., 2021). For example, in cutaneous melanoma, elevated Banfield Raftery index (lymphocyte cluster density) correlates with improved survival, while expanded Ball Hall index (infiltration spatial extent) in breast cancer associates with reduced survival (Viktor et al., 2018; Groher et al., 2019). Flagship Biosciences’ computational pathology platform analyzes PD-L1 spatial expression and generates an “Immuno-oncology Scorecard” to predict and monitor treatment response via microenvironment characterization (Caldwell et al., 2019). Conceptually, AI integrates multiple functionalities into dynamic analytical workflows, enabling holistic interpretation of tumor microenvironment composition and spatial organization. Recent computational advances analyze spatial transcriptomics to identify spatial domains, cell clusters, and molecular networks (Bin et al., 2022; Zhaoyang et al., 2022). These integrate multimodal data—histology, gene expression, spatial localization—with gene regulatory and protein interaction networks. Unlike conventional methods, stKeep uses hypergraph technology capturing cell/spot-gene-region relationships (Chunman et al., 2024). Its attention-based graph embedding projects nodes into low-dimensional space, revealing new cell states and gene associations. Thus, AI-driven dynamic modeling transcends phenomenological description, creating new opportunities for mechanistic exploration.

## 5 Challenges and future perspectives

Despite the promising potential of artificial intelligence in the fields of radiotherapy and immunotherapy, its widespread application faces several challenges. First, AI models rely on high-quality, large-scale annotated data, which is costly to produce and susceptible to subjective biases from experts. Second, differences in imaging devices and protocols among various medical institutions limit the generalizability of these models. The sensitivity of medical data also constrains data sharing, hindering the construction of large-scale datasets. Additionally, the “black box” nature of deep learning models makes their decision-making processes difficult to interpret, affecting clinicians’ trust; thus, the development of interpretable AI is crucial. To ensure clinical applicability, rigorous prospective multi-center validation of models is necessary. Therefore, the development of explainable AI is crucial. Firstly, retrospective validation should be conducted using meticulously curated large-scale datasets from multiple institutions to confirm the model’s predictive performance and generalizability. Secondly, the model’s performance and reliability in real-world clinical workflows should be assessed through prospective observational studies. Finally, prospective interventional trials should be carried out to compare AI-guided treatment decisions with standard treatment regimens, thereby evaluating their impact on clinical outcomes. On a technical level, AI can generate synthetic medical data to expand training datasets while preserving privacy, or simulate patient responses to treatment to facilitate doctor-patient communication. Adopting a distributed learning paradigm, where “data remains stationary while models move,” allows for collaborative training across institutions without sharing raw data, effectively breaking down data silos. Importantly, AI should aim to infer causal relationships from observational data to provide more reliable evidence for clinical decision-making.

## 6 Conclusion

Leveraging its inherent advantages in processing high-dimensional data, AI is emerging as a central driving force in interpreting biological complexities, predicting therapeutic efficacy, and optimizing combination strategies. Driven by interdisciplinary collaboration, the deep integration of the physical precision of radiotherapy, the biological intelligence of the immune system, and the computational power of AI is poised to offer new hopes for the cure of cancer patients.
